# Impact of HEXACO Personality Factors on Consumer Video Game Engagement: A Study on eSports

**DOI:** 10.3389/fpsyg.2020.01831

**Published:** 2020-08-05

**Authors:** Amir Z. Abbasi, Saima Nisar, Umair Rehman, Ding H. Ting

**Affiliations:** ^1^Faculty of Management Sciences, Shaheed Zulfiqar Ali Bhutto Institute of Science and Technology, Karachi, Pakistan; ^2^Department of Business Management, Karakoram International University, Gilgit, Pakistan; ^3^User Experience Design Department, Wilfrid Laurier University, Brantford, ON, Canada; ^4^Department of Management and Humanities, University of Technology Petronas, Teronoh, Malaysia

**Keywords:** consumer engagement, eSports, personality factors, HEXACO 60 items, PLS-SEM approach

## Abstract

This article aims to uncover novel insights into personality factors and consumer video game engagement modeling. This research empirically validates the role of specific HEXACO personality factors that foster consumer engagement (CE) in electronic sports (eSports) users. Using a survey-based approach, we incorporated the HEXACO 60 items and consumer video game engagement scales for data collection. Data were collected from eSports users, with 250 valid responses. WarpPLS 6.0 was used for partial least squares–structural equation modeling analyses comprising measurement and structural model assessment. The results showed that the reflective measurement model is reliable and sound, whereas the second-order formative measurement model also meets the criteria of indicator weights and collinearity values variance inflation factor (VIF). The results based on the structural model indicate that openness to experience, extraversion, agreeableness, and conscientiousness positively predict CE in eSports. This article is first among others that conceptualizes and validates the HEXACO personality traits as a reflective formative model using the hierarchical component model approach. The research model carries the explanatory capacity for CE in eSports concerning personality dimensions as indicated by the HEXACO model. It highlights the potential benefits of such research especially to marketers who could potentially employ personality modeling to develop tailored strategies to increase CE in video games.

## Introduction

### Background

Electronic sports (eSports) has become an emergent form of entertainment, with more than 380 million global viewers. Global consumer spending on video games is rapidly growing: from a total of $137.9B in 2018 to a forecasted value of $180.1B by 2021 (Pannekeet, 2018). Within gaming, competitive, tournament-based, and sport-geared video games are categorized as eSports (Jenny et al., 2017); eSports can be played real time on a myriad of platforms ranging from personal computers to gaming consoles (e.g., StarCraft II, online FIFA games (Breidbach et al., 2014; Seo and Jung, 2016). Electronic sports popularity has attracted the attention of marketers and academic scholars because of its avid-fan following (Xiao, 2019). The present research takes the first few steps toward investigating personality factors that drive consumer engagement (CE) in eSport video games.

Extensive assessment of CE in games requires unified and cross-disciplinary efforts toward understanding the relationship between users and analogous game play–related products/services (Fortes Tondello et al., 2018). Video games provide avenues for engagement where users can connect and collectively participate in multifaceted game play (Hollebeek et al., 2017). Collaborative information sharing resulting from player-to-player interaction is one of the reasons for CE (Ul Islam et al., 2017) alongside other factors that are potentially shaped by an individual’s unique temperamental attributes (Reyes et al., 2019). Research exploring how personality factors influence CE can have myriad of benefits especially from commercial standpoints; for instance, such research can aid business managers choose better market segmentation and targeting strategies based on personality-based attributes (Ul Islam et al., 2017).

Given the fact that personality is a significant factor in influencing human–computer interaction in games (Sulaiman et al., 2018), it makes sense to ascertain users’ personality characteristic in efforts to develop tailored games that drive engagement in consumer game–related interactions. “Big Five” personality attributes have been extensively investigated in previous game-focused projects and others as well (Marbach et al., 2016; Ul Islam et al., 2017; Delhove and Greitemeyer, 2018; Reyes et al., 2019), with more recent research being conducted in online-game settings (Lachlan and Maloney, 2008; Alsawaier, 2018; Sulaiman et al., 2018; Shin, 2019).

The existing research has mostly employed the Big Five personality attributes, and very limited research exists that has investigated the impact of the HEXACO personality factors on CE, especially in online video game settings. Consumer engagement is defined as “A psychological state that triggers due to two-way interactions between the consumer and video game product, i.e., eSports game, which generates a different level of consumer engagement states (cognitive, affective and behavioral)” (Abbasi et al., 2016, p. 249). As per the definition, consumer video game engagement is a higher-order formative construct that comprises three main dimensions (Abbasi et al., 2019b). Our research addresses this gap by specifically employing the fundamentally unique personality model – HEXACO, which comprises factors that include honesty–humility, emotionality, extraversion, agreeableness, conscientiousness, and openness to experience – to study the impact of personality traits on CE in eSports context. Prior studies investigating CE in video games have explored research topics such as video game addiction and scholastic achievements (Skoric et al., 2009); video games for rehabilitation (games to enhance physical therapy) (Lohse et al., 2013); mental health issues associated with video games such as stress, anxiety, and depression (Loton et al., 2016); gender differences in video game play (Jamak et al., 2018); playful-consumption experiences (Abbasi et al., 2019a, b); engagement in violent games and its link to aggressive behavior (Przybylski and Weinstein, 2019); and educational games in STEM subjects (Zuiker and Anderson, 2019). Recently, Reguera et al. (2020) have quantified engagement through playful environment, that is, video game playing.

So far, however, there is little debate on personality traits that have the potential to trigger CE in eSports environment. Hence, we cover this phenomenon in our study. This research is novel as we extend the concept of CE in eSports video games and explore the role of HEXACO personality factors in predicting CE. Besides, our study is first among others who conceptualizes and validates the HEXACO personality traits as a reflective formative model using the hierarchical component model approach.

### Rationale for Using the HEXACO Model

The most commonly used personality trait models include the Big Five model and the “five-factor” model. Both these models carry the capacity to predict individual personality traits in terms of five major personality dimensions that include conscientiousness, agreeableness, extraversion, openness to experience, and neuroticism (Costa and McCrae, 1992). In 2000, Ashton et al. (2000) conducted a study to reassess the structure of the English personality lexicon; their research comprised lexical studies of the personality structure based on approximately a dozen languages. The outcome of their research resulted in a personality model that was later categorized as HEXACO model (Ashton et al., 2000; Ashton and Lee, 2007). HEXACO-PI-R considers the six main dimensions of personality comprising of honesty–humility (H), emotionality (E), extraversion (X), agreeableness (A), conscientiousness (C), and openness to experience (O) (Lee and Ashton, 2016). Recently, Abbasi et al. (2020) emphasized that HEXACO-PI-R was better at predicting the personality differences between individuals when compared against existing personality models. HEXACO-PI-R model is akin to the Big Five model with regard to three dimensions: extraversion, conscientiousness, and openness to experience (Ashton and Lee, 2007). However, the HEXACO-PI-R model presents an additional dimension, that is, honesty–humility, and modifies the existing factors such as agreeableness and emotionality of the Big Five model. Therefore, we believe that the HEXACO model is a better substitute for existing personality models including the Big Five and the five-factor models. The benefits of choosing HEXACO over existing models are manifold. For instance, HEXACO models are established on lexical studies of personality-descriptive words in multiple languages (Lee and Ashton, 2004; Ashton et al., 2014). Also, the HEXACO model offers a more comprehensive outlook on individual personality because it has additional factors that were not accounted for in existing personality models (Worth and Book, 2014). In light of its myriad of benefits, we employ the HEXACO personality model to examine the role of personality traits that influence consumers’ engagement in eSport games.

## Hypothesis Development

### Honesty–Humility

According to Ashton and Lee (2009), honesty–humility is a unique characteristic of the HEXACO personality model. Individuals having this attribute are honest, modest, fair, and generous (Zeigler-Hill and Monica, 2015). They avoid manipulating other people for their gains. Individuals lacking this attribute are often classified as cruel, selfish, and manipulative (Andrus, 2018). To be more specific, honesty–humility is the propensity to be fair and authentic with others, even at the cost of personal suffering (Hilbig et al., 2013).

In the context of video games, individuals with pronounced personality characteristic would avoid engaging in video games. Previous research supports the notion that honesty–humility is not associated with gaming preferences (Zeigler-Hill and Monica, 2015). Honest and concerned individuals usually avoid playing video games in entirety (Worth and Book, 2014).

We thus hypothesize:

**H1: Honesty–humility has a negative association with consumer video game engagement.**

### Emotionality

An emotional individual is often sensitive, touchy, restless, and fearful (Ashton et al., 2014). Emotionality also explains an individual’s depressive tendencies and desires to seek emotional assistance (Andrus, 2018). Individual scoring high on emotionality scale are susceptible to anxiety and pain (McGrath et al., 2018).

Some studies indicate a positive relationship of emotionality with video game engagement (Worth and Book, 2014), however, in general, most studies indicate that emotional individuals avoid participating in online video games because such games can lead to disappointment or critical analysis from other players (Zeigler-Hill and Monica, 2015). According to Zeigler-Hill and Monica (2015), emotionality factor is congruent to neuroticism explained by the Big Five personality factors and is negatively associated with the daredevil preferences that are common in online video games. Personalities with elevated levels of emotionality may be uncomfortable with sensation-seeking features of daredevil preferences. A highly emotional individual often tends to avoid engaging with online video games as it involves the risk of condemnation and disapproval from others. We thus hypothesize:

**H2: Emotionality has a negative impact on consumer video game engagement.**

### Extraversion

An extravert is usually chatty, lively, dynamic, conversational, and enthusiastic (Topete, 2010). Extraverted individuals are more inclined to interact in online settings (Choi et al., 2015). According to Choi et al. (2015), extraverts are socially skillful, eager to uptake activities, and are driven to develop unique interpersonal social linkages. In the context of video games, researchers examined the positive relationship of extraversion with video game play. For example, a study related to personality and video game genres indicated a positive association of extraversion with role-playing games, action role-playing games, and real-time strategy games (Peever et al., 2012). Similarly, research suggests that progressively extraverted individuals seem to relish challenging situations often present in different game genres (Teng, 2008). Thus, we hypothesize:

**H3: Extraversion has a positive association with consumer video game engagement.**

### Agreeableness

Highly agreeable individuals tend to be relatively more trustworthy, helpful, adaptable, accommodating, and forgiving (Choi et al., 2015). Agreeableness alludes to a cohort of positive emotions toward others and often associated with approachability and friendliness (Marbach et al., 2016). On the other hand, we also regarded agreeableness as the opposite of aggressiveness and anger. In game playing, aggressiveness and anger caused annoyance among players. A gamer who is quick and temperamental usually suffers from being criticizing during game play. Players understand that it is difficult to be accepted in the eSport community if they are aggressive. Rather than having an intolerable personality and being outcast, players have chosen to be more helpful to achieve a certain goal together. The feeling of being outcast in the eSport community or in a particular group will cause a feeling of nonbelongingness; therefore, many players have prevented the development, action, or expression of aggressiveness. The suppression effect of aggressiveness leads to a higher utility in game playing.

Highly agreeable individuals care about the contentment of others and therefore would value their commitments on online platforms (Marbach et al., 2016). Furthermore, highly agreeable personalities are more likely to report higher levels of expertise, enjoyment, and control in video games (Johnson et al., 2012). We thus hypothesize:

**H4: Agreeableness has a positive association with consumer video game engagement.**

### Conscientiousness

Conscientiousness is a personality factor focused on achievement, success, discipline, accountability, and cautiousness (Choi et al., 2015). Conscientiousness personalities are cautious, well-organized, and consistent in their dealings (Topete, 2010). Such individuals perform well in professional team-based settings (Lin et al., 2001). Individuals who score high in conscientiousness tend to embrace novel experiences with vigilance (Major et al., 2006). Such individuals can competently accomplish tasks by analyzing perceived information with clarity and focus; research indicates that conscientious personalities would thrive in achievement-oriented environments such as online-game settings (Teng, 2008; Topete, 2010). Therefore, we hypothesize that:

**H5: Conscientiousness has a positive association with consumer video game engagement.**

### Openness to Experience

Individuals who are open to experience tend to be more creative, versatile, open-minded, adventurous, and in pursuit of new ideas and experiences. Such personalities actively engage in shooting games, action-oriented games, role-playing, and other similar genres (Teng, 2008; Johnson and Gardner, 2010). These personalities are receptive to different types of synthetic characters and narratives present in video games (Johnson et al., 2012). Furthermore, it has been established that a positive association exists between openness to experience and consumer video engagement (Johnson et al., 2012; Marbach et al., 2016; McGrath et al., 2018), thereby demonstrating that individuals with high openness tend to be more receptive of video games and in general more active in video game play. Thus, we hypothesize that:

**H6: Openness to experience has a positive association with consumer video game engagement.**

**FIGURE 1:**
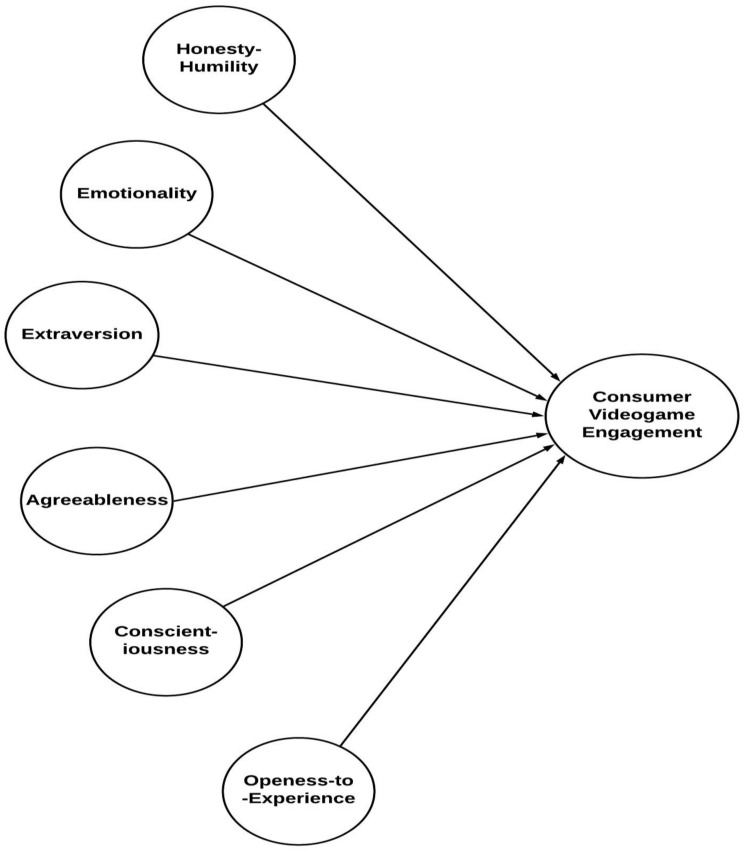
Theoretical framework.

Based on the six hypotheses above, Figure 1 portrays the relationships under study.

## Materials and Methods

### A Cross-Sectional Study

A cross-sectional survey design was implemented that allowed us to gather responses instantaneously, thereby expediting the process of data collection (Mills and Gay, 2019). Another advantage of this survey approach was that it provided us with information regarding the overall behavior of our participant population.

### Participants

This study involved teenagers aged between 14 and 19 years. Initially, data were gathered from different gaming zones in Rawalpindi and Islamabad. Furthermore, the study also collected data from teen students because this population has the highest tendency to actively engage in digital game-playing behavior (Adachi and Willoughby, 2016). Once we had a list of gaming zones located in both cities such as Rawalpindi and Islamabad, we then applied the randomizer tool to randomly select twenty gaming zones for data collection. Visiting each gaming zone, we first inquired whether eSports games such as CS Go, Call of Duty, PUBG, and so on, are being played. If the answer is yes, then we formally took the permission from the owner of a gaming zone and sought the consent from all eSports users (who were available at times of our visits), as well to formally start the data collection procedure. A questionnaire survey was administered to gather data from eSports users. To determine the required number of participants, we performed the power analysis using the G^∗^Power 3.1.9.2 (Faul et al., 2007). During the analysis, we gave the following input parameters; test family – *F*-tests; statistical test – linear multiple regression: fixed model; *R*^2^ deviation from zero, type of power analysis – *a priori*: compute required sample size – given α = 0.05, power = 0.95, and effect size = 0.15; and number of predictors = 6. Based on the input parameters, the recommended samples size was 146 [minimum required sample to perform partial least squares–structural equation modeling (PLS-SEM) analyses] with actual power = 0.950.

### Measures and Procedure for Data Collection

The questionnaire designed for this study has three major parts. The first part of the instrument is related to the demographics of respondents. It provides us with general information such as age, gender, qualification, frequency of video game play, average hours of play, genres of games played, commonly used platforms for game playing, and location where games are most frequently played.

The second part of the instrument is related to HEXACO personality factors adopted from the 60-item English version of the HEXACO-PI-R (Lee and Ashton, 2004, 2016). This part examines the six personality factors of our participant population, including honesty–humility, emotionality, openness to experience, agreeableness, extraversion, and conscientiousness.

The final part of the instrument is related to consumer video game engagement. Responses were collected regarding cognitive, affective, and behavioral engagement of the players with online video games. The scale was adapted from the previous literature, which has been formerly applied to assess consumer video game engagement (Abbasi et al., 2019a). We adapted this scale because it covers more aspects including cognitive, affective, and behavioral factors comparing the existing scales such as game engagement scale (Brockmyer et al., 2009), user engagement scale (Wiebe et al., 2014), and revised game engagement model (Procci et al., 2018). Besides, the dimensions of consumer video game engagement have achieved sufficient reliabilities and other validity tests (Abbasi et al., 2016, 2017, 2019a).

The main variables consist of the higher-order formative constructs from the HEXACO personality model (included the six personality factors). The individual personality factors were derived from associated aspects of participants’ personality. For instance, honesty–humility involves modesty, greed avoidance, sincerity, and fairness. Emotionality was captured from fearfulness, anxiety, dependence, and sentimentality. Extraversion was extrapolated from social self-esteem, social confidence, sociability, and liveliness. Agreeableness was deduced from factors such as forgiveness, gentleness, flexibility, and patience. Conscientiousness was determined from aspects such as organization, diligence, perfectionism, and prudence. The final HEXACO personality factor called openness to experience was reasoned from aesthetic appreciation, inquisitiveness, creativity, and unconventionality (Ashton and Lee, 2009).

Similarly, consumer video game engagement stemmed from a mix of cognitive, behavioral, and emotional aspects of an individual’s personality. All these three states of engagement were further elaborated: cognitive aspects were further extended into conscious attention and absorption; emotional or affective aspects were garnered from factors such as dedication and enthusiasm; and finally, behavioral engagement was surmised from factors such as social connection and interaction.

All the items in the questionnaire related to the main constructs were assessed on the Likert scales ranging from 1 to 5 (strongly disagree, disagree, neutral, agree, strongly agree).

To test the reliability of the questionnaire, a pilot study was conducted to evaluate the feasibility of the key steps, as well as to check for clarity of questions, grammatical mistakes, the feasibility of sampling technique, determining appropriate sample sizes, and reckoning overall feasibility of scale (Van Teijlingen et al., 2001). To test the reliability, we distributed 30 questionnaires to different respondents during the preliminary study.

Upon getting the response from the pilot study, we did some revisions, to ensure the correctness of the questionnaires as well as to ensure that a proper sampling protocol can be achieved. We then distributed 350 questionnaires, and 280 responses were collected. Once the data were collected, missing values and incomplete responses were identified and deleted using casewise deletion (Hair et al., 2016). As a result, 250 valid cases were left for further analysis, which also meets the minimum requirement for PLS-SEM analysis. See Table 1 for respondents’ profile.

**TABLE 1:** Shows the demographic profile of the respondents.

**Demographic analysis**	
**Respondents profile**	**Percentage %**
**Age**	
14–15	9.6
15–16	8.4
17–18	30.8
19	51.2
**Gender**	
Male	78.8
Female	21.2
**Qualification**	
SSC	11.2
Diploma/Intermediate	34
Fresh Undergraduate	28
Undergraduate	26.8
**Frequency of game playing**	
Everyday	46
Once a week	18.4
A few times a week	35.6
**Average hours of a game played**	
1–4 h	85.6
Above 4–8 h	13.6
Above 8–12 h	.8
**Most common games played**	
PUBG	58
Counter-Strike	93.6
League of Legends	86
Call of duty	84.4
Others	74.4
**The most common platform used**	
Personal computer	58
Dedicated gaming console	20.8
Smartphone	80.8
Wireless devices	97.2
Other	2.8
**Location of game playing**	
Home	76.4
Friend’s place	12.4
Cyber café	8.8
Others	13.6

### Data Analytical Approach

Partial least squares–structural equation modeling is a complete multivariate statistical investigation tool that was employed in this study to verify the study model (Hair et al., 2011). We applied the PLS-SEM approach because it can accommodate the testing of complex modeling (Hair et al., 2016, Hair et al., 2017). In addition, our study model comprised the higher-order constructs such as personality traits and consumer video game engagement. Because of the complex nature of higher-order constructs (our study involved the reflective and formative measurement models), we believe that the PLS-SEM technique can be employed for the data analyses. Moreover, our study is exploratory and based on theory development. Several studies have acknowledged that PLS-SEM is considered appropriate for exploratory studies and complex modeling involving reflective and formative constructs (Hair et al., 2017; Sarstedt et al., 2019) and theory development (Kline, 2015; Sarstedt et al., 2017). To examine the PLS-SEM analysis, our study is using the WarpPLS version 6.0, developed by Kock (2012).

## Findings

The present study followed a two-step process that is based on the measurement and structural model. First, the researcher assessed the measurement model for authenticating reliability and validity of the variables, and second, the structural model was appraised to explain the associations between the main variables.

### Step 1: Measurement Model Assessment

**FIGURE 2:**
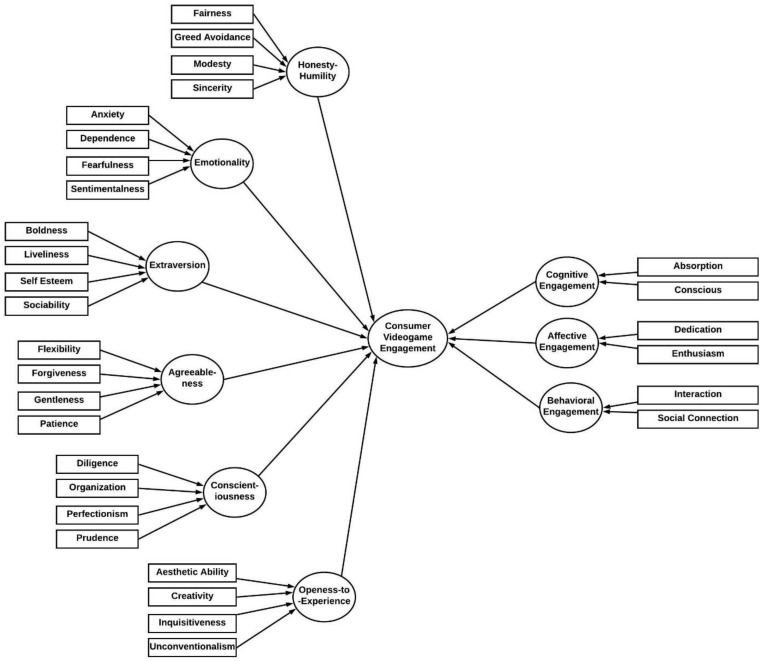
PLS-5EM model specification for measurement model assessment.

The theoretical model (Figure 1) shows the two main higher model constructs that are HEXACO personality factors and the consumer video game engagement. Figure 1 further elaborates the model into the first-order, second-order, and third-order/higher-order constructs. All the personality factors are second-order formative constructs; these are derived from the first-order reflective constructs; for example, the model illustrates that honesty–humility (second-order formative construct) is derived from fairness, greed avoidance, modesty, and sincerity (these are first-order reflective constructs). Personality characteristics are further derived from other attributes, which are stated in Figure 2 and categorized as first-order reflective or facet-level constructs for this study (Ashton et al., 2014). As explained in Figure 2, consumer video game engagement is a third-order formative construct. It is split into three main second-order formative constructs that include cognitive engagement, affective engagement, and behavioral engagement. These factors are elaborated further by first-order reflective constructs; for example, cognitive engagement is measured through conscious attention and absorption (Abbasi et al., 2017, see Figure 2).

To evaluate the reliability and validity of the model, the study first analyzes all the first-, second-, and third-order constructs in the stated order, respectively.

#### Assessment of First-Order Reflective Constructs

To assess the reliability and validity of first-order reflective constructs, the study checked three criteria such as internal consistency using Cronbach α and composite reliability (> 0.70), outer loadings (should be ≥ 0.40), convergent validity (AVE > 0.50), and discriminant validity (Sarstedt et al., 2014). The results on reflective constructs indicate that all constructs have achieved the threshold values as suggested (see Table 2).

**TABLE 2:** Assessment of measurement model.

**Scale**	**Items**	**Loadings**	***P*-value**	**CR**	**Cronbach alpha**	**Avg. variance**	**VIF**
Sincerity	Item1	0.860	<0.001	0.855	0.745	0.663	1.259
	Item2	0.768	<0.001				
	Item3	0.812	<0.001				
Fairness	Item1	0.820	<0.001	0.845	0.725	0.645	1.408
	Item2	0.775	<0.001				
	Item3	0.814	<0.001				
Greed Avoidance	Item1	0.865	<0.001	0.856	0.664	0.748	1.35
	Item2	0.865	<0.001				
Modesty	Item1	0.885	<0.001	0.879	0.724	0.784	1.192
	Item2	0.885	<0.001				
Fearfulness	Item1	0.972	<0.001	0.848	0.713	0.678	1.343
	Item2	0.971	<0.001				
	Item3	0.381	<0.001				
Anxiety	Item1	0.898	<0.001	0.893	0.759	0.806	1.266
	Item2	0.898	<0.001				
Dependence	Item1	0.887	<0.001	0.881	0.729	0.787	1.22
	Item2	0.887	<0.001				
Sentimentality	Item1	0.847	<0.001	0.851	0.737	0.657	1.244
	Item2	0.842	<0.001				
	Item3	0.738	<0.001				
Self esteem	Item1	0.787	<0.001	0.836	0.705	0.629	1.796
	Item2	0.822	<0.001				
	Item3	0.770	<0.001				
Social boldness	Item1	0.779	<0.001	0.859	0.753	0.67	1.857
	Item2	0.831	<0.001				
	Item3	0.844	<0.001				
Sociability	Item1	0.899	<0.001	0.894	0.762	0.808	0.2
	Item2	0.899	<0.001				
Liveliness	Item1	0.903	<0.001	0.898	0.773	0.815	1.628
	Item2	0.903	<0.001				
Forgiveness	Item1	0.880	<0.001	0.873	0.708	0.774	2.147
	Item2	0.880	<0.001				
Gentleness	Item1	0.741	<0.001	0.84	0.713	0.637	1.983
	Item2	0.847	<0.001				
	Item3	0.803	<0.001				
Flexibility	Item1	0.794	<0.001	0.836	0.705	0.629	2.078
	Item2	0.762	<0.001				
	Item3	0.822	<0.001				
Patience	Item1	0.885	<0.001	0.879	0.724	0.783	1.644
	Item2	0.885	<0.001				
Organization	Item1	0.889	<0.001	0.883	0.735	0.791	1.786
	Item2	0.889	<0.001				
Diligence	Item1	0.877	<0.001	0.869	0.7	0.769	1.759
	Item2	0.877	<0.001				
Perfectionism	Item1	0.731	<0.001	0.841	0.715	0.639	1.893
	Item2	0.814	<0.001				
	Item3	0.848	<0.001				
Prudence	Item1	0.816	<0.001	0.861	0.758	0.674	1.703
	Item2	0.833	<0.001				
	Item3	0.813	<0.001				
Aesthetic appreciation	Item1	0.904	<0.001	0.899	0.776	0.817	1.468
	Item2	0.904	<0.001				
Inquisitiveness	Item1	0.894	<0.001	0.888	0.748	0.799	1.956
	Item2	0.894	<0.001				
Creativity	Item1	0.810	<0.001	0.836	0.704	0.63	1.714
	Item2	0.850	<0.001				
	Item3	0.715	<0.001				
Unconventionality	Item1	0.853	<0.001	0.846	0.727	0.648	1.446
	Item2	0.792	<0.001				
	Item3	0.767	<0.001				
Conscious attention	Item1	0.713	<0.001	0.883	0.841	0.558	3.052
	Item2	0.774	<0.001				
	Item3	0.763	<0.001				
	Item4	0.764	<0.001				
	Item5	0.756	<0.001				
	Item6	0.711	<0.001				
Absorption	Item1	0.737	<0.001	0.874	0.819	0.581	3.084
	Item2	0.774	<0.001				
	Item3	0.766	<0.001				
	Item4	0.751	<0.001				
	Item5	0.781	<0.001				
Dedication	Item1	0.873	<0.001	0.895	0.846	0.641	2.188
	Item2	0.885	<0.001				
	Item3	0.874	<0.001				
	Item4	0.435	<0.001				
	Item5	0.841	<0.001				
Enthusiasm	Item1	0.877	<0.001	0.901	0.834	0.752	2.321
	Item2	0.906	<0.001				
	Item3	0.815	<0.001				
Social connection	Item1	0.816	<0.001	0.863	0.762	0.677	2.424
	Item2	0.815	<0.001				
	Item3	0.837	<0.001				
Interaction	Item1	0.731	<0.001	0.884	0.836	0.604	3.313
	Item2	0.776	<0.001				
	Item3	0.801	<0.001				
	Item4	0.831	<0.001				
	Item5	0.743	<0.001				

Table 3 shows the discriminant validity for the reflective constructs. All the diagonal values reported in the table represent the square root of the AVE of each construct. To reach discriminant validity (Fornell and Larcker, 1981), this value should be greater than its parallel correlation coefficients. In the table, all the diagonal values are greater than the off-diagonal values. Thus, discriminant validity is not an issue in this study (see Table 3).

**TABLE 3:** Discriminant validity.

	**HSin**	**Hfair**	**Hgred**	**Efear**	**Eanxity**		**Edep**	**Esenti**	**Eslfest**	**Ebold**	**Esoc**
HSin	**0.814**										
Hfair	0.201	**0.8**									
Hgreed	0.225	0.27	**0.865**								
Efearfu	0.173	0.21	0.229	**0.823**							
Enxity	0.129	0	0.201	0.187	**0.898**						
Edep	0.162	0.16	0.186	0.152	0.237		**0.887**				
Esent	0.058	0.02	0.222	0.071	0.238		0.173	**0.811**			
Eslfest	−0.03	0.09	−0.01	0.066	−0.053		0.062	0.077	**0.793**		
Ebold	0.065	0.16	0.074	0.155	0.091		0.141	0.143	0.471	**0.818**	
Esoc	0.09	0.1	0.099	0.196	0.114		0.011	0.134	0.428	0.45	**0.899**

	**ELivli**	**Aforg**	**Agent**	**Aflex**	**Apat**	**Corg**	**Cdelg**	**Cperf**	**Cprud**	**Oaest**	**Oinqu**

ELivli	**0.903**										
Aforgv	0.395	**0.88**									
Agentl	0.274	0.513	**0.798**								
Aflex	0.222	0.455	0.498	**0.793**							
Apatnc	0.202	0.425	0.36	0.455	**0.885**						
Corg	0.269	0.259	0.264	0.209	0.275	**0.889**					
Cdelig	0.206	0.263	0.123	0.19	0.333	0.456	**0.877**				
Cperf	0.217	0.261	0.226	0.316	0.338	0.469	0.447	**0.799**			
Cprud	0.177	0.136	0.133	0.352	0.261	0.316	0.304	0.428	**0.821**		
Oaesth	0.118	0.191	0.191	0.329	0.327	0.216	0.265	0.274	0.184	**0.904**	
Oinqu	0.402	0.419	0.402	0.377	0.419	0.292	0.314	0.397	0.256	0.392	**0.894**

	**Creat**	**Unc**		**ConAt**	**Asorp**	**Dedic**		**Enthu**		**Socon**	**Interc**

Creatit	**0.794**										
Uncon	0.29	**0.805**									
ConAte	0.298	0.239		**0.747**							
Asorp	0.327	0.14		0.65	**0.762**						
Dedic	0.248	0.189		0.575	0.643	**0.801**					
Enthu	0.283	0.162		0.598	0.619	0.559		**0.867**			
Socon	0.277	0.206		0.621	0.65	0.483		0.533		**0.823**	
Interac	0.306	0.224		0.701	0.652	0.642		0.688		0.637	**0.777**

*Square roots of average variances extracted (AVEs) shown on diagonal.*

#### Assessment of Second-Order Formative Constructs

To assess second-order formative constructs, a two-stage method was adopted (Becker et al., 2012). To find the validity of the second-order formative construct, variance inflation factor (VIF) of all the items must be assessed, and the value should be less than five as recommended by Hair et al. (2011) or 3.3 as recommended by Kock (2017). Hair et al. (2011) also emphasized that the construct’s weight and significance level must be assessed. The value of the significance level must be less than 0.05. Table 4 reveals the significance or *P*-value of indicator weights associated with second-order formative constructs and VIF of the variables; these values in Table 4 match the discussed threshold criteria. Hence, our second-order formative constructs are valid and reliable for further analysis (see Table 4).

**TABLE 4:** Assessment of the measurement model on second-order formative constructs (e.g., honesty-humility, emotionality, and etc.).

**Constructs**	**Items**	**Scale type**	**Weights**	**Sig**	**Full collinearity**	**VIF**
Honesty-humility		Formative			1.153	
	Sincerity		0.452	<0.001		1.08
	Fairness		0.48	<0.001		1.136
	Greed avoidance		0.499	<0.001		1.134
Emotionality		Formative			1.166	
	Fearfulness		0.33	<0.001		1.05
	Anxiety		0.466	<0.001		1.132
	Dependence		0.42	<0.001		1.089
	Sentimentality		0.383	<0.001		1.077
Extraversion		Formative			1.7	
	Social self esteem		0.323	<0.001		1.402
	Social boldness		0.338	<0.001		1.473
	Sociability		0.346	<0.001		1.527
	Liveliness		0.317	<0.001		1.376
Agreeableness		Formative			2.007	
	Forgiveness		0.333	<0.001		1.545
	Gentleness		0.33	<0.001		1.552
	Flexibility		0.335	<0.001		1.557
	Patience		0.304	<0.001		1.372
Conscientiousness		Formative			1.526	
	Organization		0.343	<0.001		1.436
	Diligence		0.336	<0.001		1.398
	Perfectionism		0.361	<0.001		1.54
	Prudence		0.3	<0.001		1.263
Openness-to-experience		Formative			1.787	
	Aesthetic app		0.347	<0.001		1.286
	Inquisitiveness		0.373	<0.001		1.396
	Creativity		0.364	<0.001		1.361
	Unconventionality		0.303	<0.001		1.172
Cognitive engagement		Formative			3.470	
	Conscious attention		0.551	<0.001		1.731
	Absorption		0.551	<0.001		1.731
Affective engagement		Formative			2.673	
	Dedication		0.566	<0.001		1.454
	Enthusiasm		0.566	<0.001		1.454
Behavioral engagement		Formative			3.224	
	Social connection		0.553	<0.001		1.682
	Interaction		0.553	<0.001		1.682

#### Assessment of Third-Order/Higher-Order Formative Construct

Again, to assess the validity of the third-order construct, that is consumer video game engagement, the study used WarpPLS version 6.0. Initially, the value of VIF was assessed, and then the significance level of the indicator’s weight was checked. Table 5 shows the values of VIF, indicator weights, and their significance level. All the values of each construct have VIF below five, and associated indicator weights meet the significance level except the affective engagement. Under such situation, Hair et al. (2016) recommended to assess the outer loading of the item, and if the outer loadings exceed the value of 0.40, then we can keep an item. Following the guideline, we examined the outer loading for affective engagement and found that it exceeded the critical value of 0.40. Therefore, these values confirm the validity of the third-order formative construct also (see Table 5).

**TABLE 5:** Assessment of the measurement model of higher-order formative construct (consumer videogame engagement).

**Constructs**	**Items**	**Scale type**	**Weights**	**Sig**	**Full Collinearity**	**VIF**
Consumer VGE		Formative			1.549	
	Cognitive Eng		0.468	<0.001		3.360
	Affective Eng		0.072	0.125		2.644
	Behavioral Eng		0.526	<0.001		3.089

### Step 2: Structural Model Assessment

The study used WarpPLS version 6.0 to check the framework model and hypotheses. For this, we assessed the value of path coefficient with effect size and *T*-value and the significance of the *R*^2^ coefficient. Effect size measures the impact of the independent variable on the dependent variable. According to the values of the effect size given in Table 6, we conclude the following:

**TABLE 6:** Assessment of the structural model.

**Hypothesis testing**		**Path coefficient**	**SE**	**F2**	***T*-value**	***P-*value**	**Result**
H1: Honesty-humility	Con VGE	0.065	0.063	0.012	1.03	0.15	Not supported
H2: Emotionality	Con VGE	0.07	0.062	0.014	1.12	0.132	Not supported
H3: Extraversion	Con VGE	0.145	0.062	0.067	2.33	0.01	Supported
H4: Agreeableness	Con VGE	0.232	0.061	0.115	3.8	<0.001	Supported
H5: Conscientiousness	Con VGE	0.184	0.061	0.08	2.87	0.002	Supported
H6: Openness to Exp	Con VGE	0.177	0.061	0.084	2.9	0.002	Supported

item Players’ honesty–humility and emotionality factors have no effect on predicting consumer video game engagement.
item In contrast, players’ conscientiousness, openness to experience, agreeableness, and extraversion factors have more than a small effect on developing consumer video game engagement. Hence, our proposed hypotheses are accepted.

**FIGURE 3:**
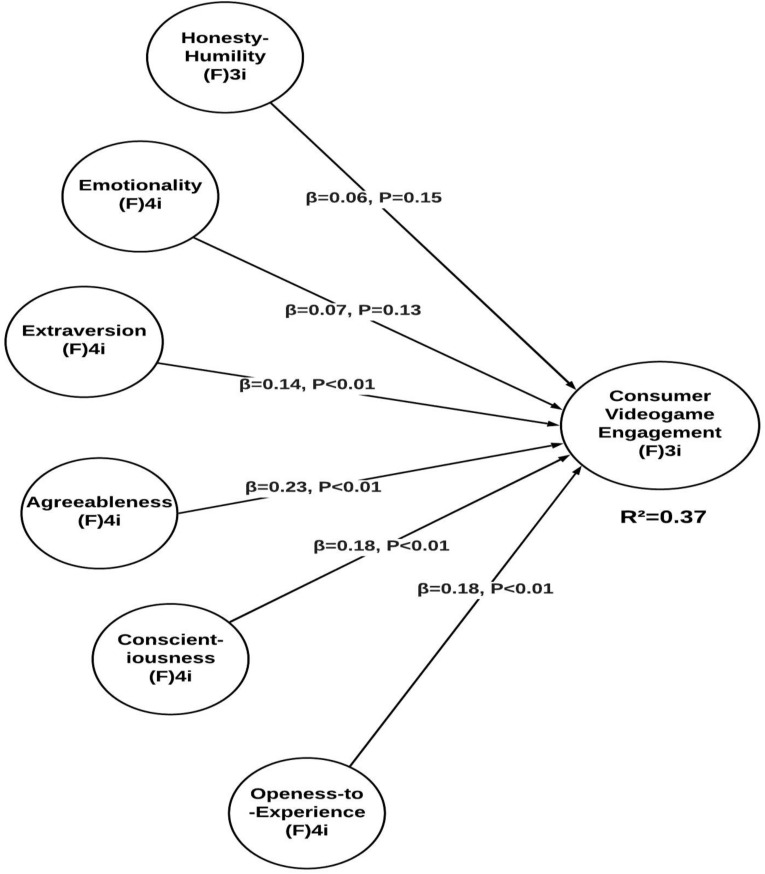
Structural model assessment.

In addition to the effect size, we also calculated the *P*-value, *T*-value, and path coefficient for our study hypotheses. The results shown in Table 6 illustrated that honesty–humility has an insignificant relationship with consumer video game engagement (path = 0.065; *T* = 1.03; *P* = 0.15) – H1 is not accepted. Furthermore, the path coefficient, *T*-value, and *P*-value for depicting the relationship between emotionality and consumer video game engagement are 0.07, 1.12, and 0.132, respectively. Because this does not meet the set criteria, our second hypothesis is also rejected. This means that there is no significant relationship between emotionality and consumer video game engagement – hence, H2 is not supported. Extraversion has a significant relationship with consumer video game engagement with a path coefficient of 0.145, *T*-value of 2.33, and *P*-value of 0.01—and as a result, H3 is accepted. Similarly, agreeableness, conscientiousness, and openness to experience have path coefficient values of 0.232, 0.184, and 0.177 and *T*-values of 3.8, 2.87, and 2.9, respectively. Also, the *P*-values shown in the table are < 0.001, 0.002, and 0.002 accordingly – therefore, H4, H5, and H6 are accepted. See Table 6 and Figure 3 for more details.

In addition, we examined the correlations between the personality traits, and the results showed that there is no high correlation issue. See Table 7 for more details.

**TABLE 7:** Correlations matrix using HEXACO 60-item English version.

	**H**	**E**	**X**	**A**	**C**	**O**
Honesty-humility	1					
Emotionality	0.324	1				
Extraversion	0.078	0.209	1			
Agreeableness	−0.033	0.121	0.580	1		
Conscientiousness	0.019	0.036	0.385	0.429	1	
Openness to experience	0.092	0.104	0.419	0.544	0.546	1

## Discussion

With the addition of different gaming platforms, eSport video game is rapidly gaining prominence in the gaming industry. This study employs the HEXACO personality model to establish a relationship between consumer personality and consumer video game engagement in the context of eSports. Quantitative methods were employed in this research, and HEXACO-PI-R 60 items were used to investigate the personalities of consumers engaged in eSports. The study empirically tested and validated the proposed model using WarpPLS version 6.0 for SEM analysis. This research presents novel insights in uncovering the specific personality factors that drive consumers’ video game engagement.

According to the data analysis, honesty–humility, and emotionality factors carry an insignificant impact on consumer video game engagement, whereas extraversion, agreeableness, consciousness, and openness to experience have a significant effect on consumer video game engagement.

As mentioned earlier, our first hypothesis indicates that honesty–humility has an insignificant impact on consumer video game engagement. Previously, Worth and Book (2014) also empirically tested this premise; they demonstrated that personalities covering the characteristic are less inclined to engage in player-versus-player–style games. Furthermore, games that involve profit manipulation, rule breaking, and material gain are also correlated with a low characteristic of honesty–humility (Andrus, 2018). Insignificant association of this attribute with consumer video game engagement is also demonstrated by Zeigler-Hill and Monica (2015). Games involve exploitation and strategic maneuvering, which can be less appealing for individuals who score high on honesty–humility.

Our second hypothesis revealed an insignificant relationship of emotionality with consumer video game engagement. In prior studies, a negative relationship was also confirmed between emotionality and daredevil preferences (Zeigler-Hill and Monica, 2015). It is important to highlight that emotional individuals prefer to avoid challenging scenarios where there is a likelihood to receive negative feedback and social disapproval. Furthermore, emotional individuals tend to demonstrate dour outlooks, which can aggravate in gaming contexts. Therefore, it is not surprising that emotionality factor does not indicate a positive association with consumer video game engagement.

Our third hypothesis of the study showed a positive relationship of extraversion with consumer video game engagement. Previous literature also confirms the presence of an insignificant relation in the context of player game preferences (Andrus, 2018), video game preferences (Zeigler-Hill and Monica, 2015), and game-playing style (Bean and Groth-Marnat, 2016). Generally, personalities that are social, optimistic, and confident actively engage in video games because gaming environments appeal to their individual psychosomatic inclinations.

Our fourth hypothesis shows a positive relationship between agreeableness and consumer video game engagement. In previous research, agreeableness dimension carries a positive correlation in multiplayer games environment and “helping” style games (Worth and Book, 2014), as well as a positive correlation with a preference to play challenging games (Zeigler-Hill and Monica, 2015). So, individuals with this attribute are adaptable and understanding and carry the proclivity to engage in games for social rapport or entertainment purposes actively.

Our fifth hypothesis shows that consciousness has a positive association with consumer video game engagement. In previous research, conscious individuals have demonstrated achievement-oriented behaviors in game-based settings (Zeigler-Hill and Monica, 2015). Zeigler-Hill and Monica (2015) have indicated that individuals with high consciousness scores prefer games that involve accomplishing arduous tasks or solving challenges rather than indulging in game play purely for leisure purposes. Therefore, from our study, we can conclude that well-organized, disciplined, and careful individuals prefer to invest time in experiencing different genres of thought-provoking games.

Our final hypothesis shows a positive relationship between openness to experience with consumer video game engagement. Literature confirms the same relationship: for instance, a study revealed that online-game players are higher in openness to experience than nonplayers (Teng, 2008). Also, openness to experience is associated with the gratification of play and shows the highest positive association for unique game behavior predilections (Bean and Groth-Marnat, 2016; Andrus, 2018). Therefore, we can say that individuals with openness to experience are eager to seek new information and are creative, imaginative, and adaptable; the presence of such psychographics results in a greater drive for engagement in video games.

## Implications and Further Research

### Theoretical Implications

This study makes several theoretical contributions. First, we present an empirical study of the HEXACO personality model and its association with consumer video game engagement in the context of eSports. Previous literature added that Big Five personality dimensions carry an impact on CE in the context of online brand communities such as social media platforms (Ul Islam et al., 2017). However, we extend the existing literature on personality traits, especially focusing on video game studies through investigating a novel model, that is, HEAXCO in the realm of consumer behavior and eSports settings. We demonstrate that certain dimensions of the HEXACO model contribute to driving CE in eSports. Second, this study also adds value to the current gaming research within the marketing literature. This research can aid researchers and marketers that are interested in analyzing empirical work that investigates CE with the video game industry. Third, we advance the earlier studies on personality traits through applying the hierarchical component model approach (Becker et al., 2012; Sarstedt et al., 2019) to establish and validate higher-order constructs. Fourth, we contribute to the notion of consumer video game engagement as we provide the evidence that personality traits do impact on CE in eSports context.

### Managerial Implications

This study also makes critical managerial contributions. First, this article highlights how marketers can capitalize on consumers’ personality factors by focusing their investments on specific personality attributes that are predicted to optimize video game engagement. Secondly, our model offers marketing practitioners the opportunity to develop video game strategies based on their target consumers’ personality factors and their expected effect on CE, which are extremely substantial in today’s era of one-to-one marketing and big data analytics. Third, video game developers can also develop specific games by capturing consumer’s interest according to each personality factor; thus, ultimately, their market share and overall growth in the industry can be maximized. A clearer picture of consumers’ personality characteristics may also help practitioners garner a better understanding of how to strategically build a process to engage customers in video game settings actively.

### Future Research

Despite its contributions, this study is still in its exploratory stage to understand the personality factors and consumer video game engagement and therefore subjected to several limitations. The first limitation is on the assumption that gamers and eSport gamers are assumed to take on the role as what is observed. With the six attributes that we have identified, we have taken the eSport gamers and personalities on the face value. We believe that it is also important to understand what takes place throughout the development of the attitude and behavior of these gamers. This could be done by using a longitudinal study (development of behavior through a process of sampling different sample groups) or conducting an experiment on the personality traits that are captured in the HEXACO 60 items. In experimental studies, control groups should be able to mobilize to capture the effects of the personalities. Second, to validate the HEXACO 60 items, the sample size is relatively small and focused on respondents from Pakistan. For a better generalization, there should be efforts to collect more samples, not only within a country but also to simultaneously expand the data collection to different countries (to capture the differences in cultures as well). Third, our study is limited in terms of the scope of its investigation within the context of eSports, whereas this study can also be extended to other genres of video games including intellectual games or virtual reality games and to investigate how consumers’ personality characteristics predict consumers’ preferred game-product preferences. Fourthly, with the advent of eSport gaming, games are not only played by men, but also by women. We acknowledge the unbalanced gender distribution in our study. Care should be taken to include a better representation of gender distribution in future studies. The condition of nongamers versus gamers (or occasional gamers) should also be defined, to understand and capture the unprecedented conditions and personality differences.

## Data Availability Statement

The datasets generated for this study are available on request to the corresponding author.

## Ethics Statement

The studies involving human participants were reviewed and approved by the Departmental Ethical Review Committee Shaheed Zulfikar Ali Bhutto Institute of Science and Technology (Szabist). The patients/participants provided their written informed consent to participate in this study.

## Author Contributions

AA, SN, and UR worked on the idea development and conceptual design. SN and AA worked on the data analyses. SN, UR, AA, and DT edited the manuscript and improved the contents of the manuscript. All authors contributed to the article and approved the submitted version.

## Conflict of Interest

The authors declare that the research was conducted in the absence of any commercial or financial relationships that could be construed as a potential conflict of interest.
